# MiR-106b induces cell radioresistance via the PTEN/PI3K/AKT pathways and p21 in colorectal cancer

**DOI:** 10.1186/s12967-015-0592-z

**Published:** 2015-08-04

**Authors:** Lin Zheng, Yuqin Zhang, Yan Liu, Min Zhou, Yanxia Lu, Li Yuan, Chao Zhang, Min Hong, Shuang Wang, Xuenong Li

**Affiliations:** Department of Pathology, School of Basic Medical Sciences, Southern Medical University, Guangzhou, 510515 Guangdong Province China; Department of Pathology, Nanfang Hospital, Southern Medical University, Guangzhou, 510515 Guangdong Province China; Department of Oncology, The First Affiliated Hospital of Jinan University, Guangzhou, 510515 Guangdong Province China; Department of Pathology, Sun Yat-Sen University Cancer Center, Guangzhou, 510515 Guangdong Province China

**Keywords:** Colorectal cancer, MiR-106b, Radioresistance, Stemness, PTEN, p21

## Abstract

**Background:**

Radioresistance is a challenge in the treatment of patients with colorectal cancer (CRC). Individuals display different therapeutic responses to preoperative radiotherapy, and the need of targeted therapies is urgent. MicroRNAs (miRNAs) are involved in essential biological activities, including chemoresistance and radioresistance. Several research studies have indicated that miRNA played an important role in sensitizing cells to ionizing radiation (IR). MiR-106b, a member of the miR-106b-25 cluster, is frequently dysregulated in many human cancers, including CRC. However, the function of miR-106b in radioresistance is currently poorly understood.

**Methods:**

A series of in vitro and in vivo studies were performed to investigate the roles of miR-106b on cell radioresistance in CRC.

**Results:**

We found overexpression of miR-106b could induce resistance to IR in vitro and in vivo in SW620 cells. Correspondingly, knocking down miR-106b in SW480 yielded the opposite effect. In addition, overexpression of miR-106b could enhance the tumour-initiating cell capacity without or with IR condition, such as the colony sphere formation capacity and the upregulation of stemness-related genes (CD133, Sox2). We further identified PTEN and p21 as novel direct targets of miR-106b by using target prediction algorithms and a luciferase assay. Overexpression of miR-106b reduced the expression of PTEN and p21 and increased the expression of p-AKT, which is a downstream of PTEN. Restoring the expression of PTEN or p21 in stably miR-106b-overexpressed cells could rescue the effect of miR-106b on cell radioresistance. Together, the acquisition of tumour-initiating cell capacity endowed CRC cells with the potential of resistance to irradiation.

**Conclusions:**

These observations illustrated that miR-106b could induce cell radioresistance by directly targeting PTEN and p21, this process was accompanied by tumour-initiating cell capacity enhancement, which is universally confirmed to be associated with radioresistance. Our data suggested that miR-106b at least partly induces cell radioresistance in CRC.

**Electronic supplementary material:**

The online version of this article (doi:10.1186/s12967-015-0592-z) contains supplementary material, which is available to authorized users.

## Background

Colorectal cancer (CRC) is one of the most common human malignant tumours worldwide. Surgery is the main treatment for patients with colorectal cancer. After radical surgery, approximately 50% CRC cases experience recurrence and metastasis. CRC, specifically rectal cancer, frequently presents at a locally advanced stage. A short-term regimen of high-dose preoperative radiotherapy reduces the rate of local recurrence and improves survival among patients with resectable rectal cancer [1]. Thus, increasing attention should be paid to pre-operative radiotherapy. However, the therapeutic response to preoperative radiotherapy differs among patients [2]. Non-responders, who minimally benefit from preoperative radiotherapy, only suffered toxic effects and perhaps missed the best treatment opportunity, which suggests that promoting the sensitivity of CRC to radiotherapy is essential.

MicroRNAs are a family of small noncoding RNAs that regulate gene expression via special sites within the 3-untranslated Regions (3′UTR) of target-mRNAs [3–6]. Mounting evidence suggests that miRNAs affect the pathogenesis classification, diagnosis, prognosis and progression of cancer [7, 8].

MiRNAs have also been implicated in the radiotherapy response of some tumours. Lin28-let7 modulates the radiosensitivity of human cancer cells via the activation of K-Ras [9]. miR-9 and let-7g could enhance the sensitivity to ionizing radiation by suppressing NFkappaB1 [10]. MicroRNA-181a sensitizes human malignant glioma U87MG cells to radiation by targeting Bcl-2 [11]. Up-regulating miR-101 efficiently reduced the protein levels of DNA-PKcs and ATM in these tumour cells and most importantly, sensitized the tumour cells to radiation in vitro and in vivo by targeting DNA-PKcs and ATM [12].

Tumour-initiating cells have been proposed to be responsible for the resistance to radiation, as already evidenced in cancer [13, 14]. MiR-106b, as a member of the miR-106b-25 cluster, is known to promote cancer cell proliferation and survival in gastric cancer and hepatocellular carcinoma [15–17]. Brett et al. found that miR-106b~25 regulates neural stem/progenitor cells (NSPC) via the insulin/IGF-FoxO pathway, which may have important implications in the homeostasis of the neural stem cell (NSC) pool during aging [18]. However, the relationship between miR-106b, stemness and radioresistance has not yet been elucidated.

A series of in vitro and in vivo studies demonstrated that the ectopic expression of miR-106b could promote cell proliferation and tumour growth, enhance the tumour-initiating cell capacity and induce cell resistance to ionizing radiation in colorectal cancer. The results revealed the novel roles of miR-106b in colorectal cancer and suggested that miR-106b may be a candidate for the treatment of patients with CRC.

## Methods

### Clinical specimens

Colorectal cancer tissues were collected from fresh surgical specimens, frozen in liquid nitrogen, and stored at −80°C until further analysis. All tissues had been histologically confirmed to be adenocarcinoma. Pathologic verification and classification were performed based on the system of the International Union Against Cancer. The research protocol was approved by the Ethics Committee at Nanfang Hospital, and written consent was obtained from all patients for the use of their tissues.

### Cell culture

Human colonic carcinoma cell lines HT29, SW480, SW620 and LOVO were obtained from American Type Culture Collection (ATCC). The CRC cell lines were cultured in RPMI-1640 medium (Gibco, USA) supplemented with 10% fetal bovine serum (FBS; Gibco, USA), 100 IU/ml penicillin and 100 g/ml streptomycin in humidified 5% CO2 at 37°C.

### RNA isolation, reverse transcription, and qRT-PCR

The total RNA was extracted from CRC cell lines with RNAiso Plus (Takara, Japan). To detect miR-106b, a stem-loop reverse transcription-polymerase chain reaction (RT-PCR) was performed using All-in-One TM miRNA quantitative RT-PCR (qRT-PCR). The sequence-specific forward primers for mature miR-106b and the U6 internal control were 5-TGTAAAGTGCTGACAGTGCA-3 and 5-CTCGCTTCGGCAGCACA-3, respectively. The relative expression was calculated via the comparative cycle threshold (Ct) method using the expression of U6 small nuclear RNA as the reference. The qRT-PCR for the analysis of mRNA expression was performed on a Stratagene ABI PRISM7500 Fast Real-time PCR system using the SYBR Green qRT-PCR master mix (TaKaRa) and GAPDH as an internal control. The primers are listed in Additional file 1: Table S1. All Data were processed using the 2^−△△CT^ method.

### MicroRNA mimics, siRNA transient transfection

MicroRNA-106b mimics (sense 5′-UAAAGUGCUGACAGUACAGUGCAGAU-3′ and anti-sense 5′-AUUUCACGACUGUCACGACUA-3′), miR-106b inhibitor (5′-AUUUCACGACUGUCACGACUA-3′), the negative control (5′-CAGUACUUUGUGUAGUACAA-3′), PTEN-shRNA (sense 5′-GAGCGUGCAGAUAAUGACAdTdA-3′ and anti-sense 3′-dAdT CUCGCACGUCUAUUACUGU-5′) and p21-shRNA (sense 5′-GAAAUAAACGGGACUGAAA dTdT-3′ and anti-sense 3′-dTdTCUUUAUUUGCCCUGACUUU-5′) were purchased from GenePharma (Shanghai, China). The transfection was performed using Lipofectamine™ 2000 (Invitrogen, USA) according to the instructions provided by the manufacturer. Twenty-four or 48 h after transfection, the cells were harvested for further experiments.

### Construction of plasmid vectors and transfection

The coding region of PTEN and p21 was PCR-amplified from human genomic DNA using primer pairs (PTEN primers forward, 5′-aaggatccCCAGACATGACAGCCATCATC-3′ and reverse 5′-cacaactcgagTCAGACTTTTGTAATTTGTGTATGC-3′ and p21 primers forward, 5′-aaggatccGGCGCCATGTCAGAACCGGCTGGGGATGT-3′ and reverse 5′-cacaactcgagTTAGGGCTTCCTCTTGGAGAAGATCAG-3′), digested with BamHI and XhoI and ligated to the pcDNA3.1 vector (Additional file 2). The transfection was performed using Lipofectamine™2000 (Invitrogen, USA) according to the instructions provided by the manufacturer.

### Viral vectors

The Viral vectors were purchased from GENECHEM company, Shanghai, China (Additional file 3).

### Dual-luciferase reporter assay

The sequences of 3′UTR PTEN and were PCR-amplified from human genomic DNA using primer pairs (PTEN primers forward, 5′-cacaactcgagTGGCAATAGGACATTGTGTCA-3′ and reverse 5′-aaggatccAACAACAAGCAGTGACAGCG-3′ and p21 primers forward, 5′-cacaagtcgacTCCGCCCACAGGAAGCCTGCAGTCC-3′ and reverse 5′-aaggatccTTACAAGTAAAGTCACTAAGAATCA-3′), digested with BamHI and XhoI and ligated to the pLuc vector. They were then cloned downstream of the Firefly luciferase stop codon in the pLuc control vector (Promega). All constructs were verified by DNA sequencing (Additional file 4). Cells were seeded in 48-well plates. After 24 h of incubation, the cells were co-transfected with 1 mg of 3′UTR-PTEN or with 3′UTR mut-PTEN combined with the control oligonucleotide (final concentration of 80 nM), mimics (80 nM) or inhibitor (80 nM) using Lipofectamine 2000 (Invitrogen) according to the manufacturer’s protocol. Forty-eight hours after transfection, the Luciferase activity was measured using the Dual Luciferase Reporter Assay System (Promega). All transfection experiments were conducted in triplicate and repeated independently 3 times.

### MTT assay

The viable cell numbers were measured with a 3-(4,5-dimethylthiazol-2-yl)-2,5-diphenyltetrazolium bromide (MTT) assay. The cells were plated in 96-well plates and incubated. Twenty microlitres of 5 mg/mL MTT (Sigma, USA) was added to each corresponding test well, and the mixture was incubated for 2 h in a 37°C incubator. The supernatant was then discarded, and 100 μl of DMSO (dimethyl sulphoxide) was added to each well to dissolve the formazan. The optical density (OD) was evaluated by measuring the absorbance at 450 nm of each well, which was read on a spectrophotometer.

### Survival foci formation assay

A predetermined number of cells were seeded in 6-well culture plates, and the cells were then incubated for 24 h to allow settling. The cells were treated with a range of IR doses [0, 2, 4, 6 and 8 Gy, Nasatron (Cs-137) irradiator]. After incubation at 37°C for 14 days, the cells were washed twice with PBS and stained with Giemsa solution. The number of colonies that contained ≥50 cells was counted under a microscope using the following formula: plate clone formation efficiency = (number of colonies/number of cells inoculated) × 100%. The survival fractions (SF) were calculated by normalizing the data to the plating efficiency of appropriate control groups. We used GraphPad Prism (GraphPad Software, LaJolla, CA, USA) to fit the cell survival curve in accordance with a standard linear-quadratic (LQ) model and obtain the values of the survival fraction of a range of IR doses.

### Flow cytometric analysis of apoptosis

One million cells were harvested and washed twice with cold PBS, fixed in ice-cold 70% ethanol, and incubated overnight at −20°C. The cells were then stained with 40 μg/ml of propidiumiodide (PI) for 30 min. The cells were collected and analysed with the Cell Quest software (Becton–Dickinson Co, NJ, USA). The percentage of cells with apoptotic nuclei (% apoptosis) was calculated. Each experiment was performed in triplicate.

### Cell-cycle analysis

The cells were harvested, washed twice with cold PBS and fixed overnight at 4°C in 70% ethanol. After the cells were washed twice with PBS, their DNA was stained with the Cell Cycle Detection Kit (KeyGen, Nanjin, China). The samples were quantified by flow cytometry (Becton–Dickinson, NJ, USA) and results were analysed with the Modfit LT software (Verity Software House, Topsham, ME, USA) according to the manufacturer’s instructions.

### Tumour sphere formation assay

The cells were digested with 0.25% trypsin (Sigma, St. Louis, MO, USA), washed twice with calcium/magnesium-free PBS, suspended in sphere formation medium (DMEM-F12 50 ml + 100 g/ml EGF + 100 g/ml bFGF + B27 supplement 1 ml), and seeded in 6-cm or 6-well plates (3,000 cells/ml). The cells were cultivated for 5–7 days (depending on the cell type), and the spheres were then counted under a microscope.

### Immunofluorescence

The cells were seeded on cover slips overnight, fixed with 4% paraformaldehyde for 30 min and treated with 0.25% Triton X-100 for 15 min. After blocking in 10% normal blocking serum at room temperature for 10 min, the slides were incubated with antibodies at 4°C overnight followed by washing with PBS three times. The cover slips were then incubated with Texas Red (TR)-conjugated antibodies for 30 min at room temperature, followed by staining with 6-diamidino-2-phenylindole (DAPI; Invitrogen).

### Protein isolation and western blotting

The cells were washed twice with cold phosphate-buffered saline (PBS) and lysed on ice in RIPA buffer with 1% PMSF (KeyGen, Nanjin, China). The protein lysates were resolved on 10% SDS polyacrylamide gels, transferred to PVDF membranes and blocked in 0.1% Tween20 and 5% skim milk protein in Tris-buffered saline. The membrane was incubated with primary antibodies (E-cadherin, Vimentin, CD133, Sox2, p21, PTEN, AKT, p-AKT and γ-H2AX purchased from Epitomics company) followed by incubation with HRP-labelled rabbit IgG, and the proteins were detected via chemiluminescence. The membrane was then washed and visualized with horseradish peroxidase (HRP)-conjugated secondary antibodies for 1 h. The signals were detected with enhanced chemiluminescence (KeyGen, Nanjin, China).

### In vivo tumour growth and xenograft tumour radiosensitivity assay

Athymic nude mice aged 4–6 weeks (GuangDong Experimental Animal Centre) were used for tumour implantation. All animal experiments strictly adhered to the Regulations for the Administration of Affairs Concerning Experimental Animals outlined in the Chinese national guideline for animal experiments issued in 1988. All procedures that involved animals and their care in this study were approved and performed by the Southern Medical University Institutional Animal Care and Use Committee (Permit Number: 44007200000784). The cells were harvested by trypsinisation, washed twice with cold serum-free medium, and re-suspended with 200 μl serum-free medium. For the xenograft tumour assay, 2 × 10^6^ cells were subcutaneously injected into the backs of nude mice. After tumours were detected, the tumour size was measured with a slide calliper, and the tumour volume was determined with the following formula: 1/2 × length × width^2^. Each group consisted of six athymic nude mice, and the mean tumour volume ± SD of each group was calculated. The harvested tumours were imaged immediately after sacrifice. To evaluate tumour radioresistance in vivo, the mice were exposed to 8 Gy X-rays when the tumours reached an average volume of approximately 200 mm^3^. The tumour inhibition rate was recorded and calculated every 4 days: Inhibition rate = (1 − irradiation treatment group/control group) × 100%.

### Immunohistochemistry

After deparaffinisation and rehydration, the slides were incubated with 3% hydrogen peroxide solution for 10 min. After a washing procedure with the supplied buffer, the tissue sections were repaired for 40 min with ethylenediamine tetraacetic acid. The slides were again incubated with the primary antibody overnight at 4°C. The primary polyclonal rabbit antibody (Bcl-2 and Bax, Bioword) was diluted at 1:100. The slides were then incubated with the secondary antibodies (anti-rabbit, DakoCytomation). The tissue staining was visualized with DAB (DakoCytomation). The slides were counterstained with haematoxylin and dehydrated. Phosphate buffered solution (PBS) was used as the primary antibody for the negative controls. Two pathologists each reviewed the results of immunostaining.

### Statistical analysis

Student’s t test and one-way ANOVA analysis were performed to assess statistical significance. Spearman’s correlation coefficient was calculated to test the association between miR-106b and PTEN in the classes normal versus tumour. The results of all experiments are expressed as the mean ± standard deviation SD of 3 independent experiments. P values<0.05 were considered statistically significant.

## Results

### MiR-106b induces CRC cell resistance to irradiation

**Fig. 1:**
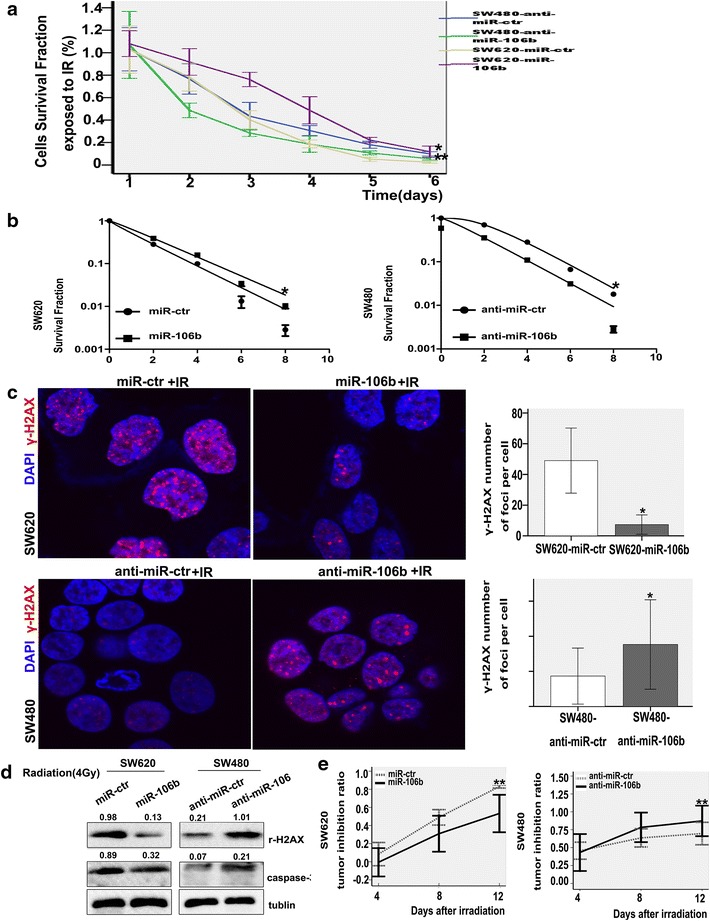
MiR-106b enhances the cell radioresistance. **a** The detection of cell sensitivity to irradiation by MTT after miR-106b overexpression or downregulation. Data shown are the mean and SE from three independent experiments.*p<0.05 **p<0.01. **b** Impact of miR-106b on cell survival foci formation when exposed to irradiation (2, 4, 6, 8 Gy). The cell survival curve of the clonogenic assay was obtained using the L-Q Linearity Quadri-model.*p<0.05. **c** Effect of miR-106b on DNA damage detected by immunofluorescence when exposed to radiation (4 Gy, 6 h). The γ-H2AX staining is shown in the *left panels* and the numbers of γ-H2AX foci are shown in the *right panels*.*p<0.05. **d** γ-H2AX and caspase-3 were examined by western blot when cells were exposed to radiation (4 Gy, 48 h). **e** Effects of miR-106b on the xenograft radiosensitivity. Tumour sizes were measured at different time points until the mice were sacrificed. The tumour inhibition ratio was calculated every 4 days after exposure to radiation (8 Gy). **p<0.01.

First, we analysed the expression pattern of miR-106b in CRC cell lines with different degrees of differentiation, including LOVO (undifferentiated), HT-29 (highly differentiated), SW620 (poorly differentiated), and SW480 (highly differentiated) cells. We found that the expression of miR-106b was higher in the highly differentiated cells lines HT-29 and SW480 (Additional file 5: Figure S1A). Cell lines that stably overexpressed miR-106b or expressed reduced amounts of miR-106b were established via lentiviral transduction, and this procedure is shown in Additional file 5: Figure S1B (*p<0.05). MTT assays were carried out to evaluate the radiosensitivity when cells were exposed to X-rays at a dose of 4 Gy. The data indicated that miR-106b could enhance the cell radioresistance (*p<0.05 **p<0.01; Fig. 1a). The colony survival assay is considered a canonical standard to determine radiosensitivity. Thus, we sought to explore the effect of miR-106b on the colony survival of CRC cells in the presence of ionizing radiation. The cells were irradiated with various doses of X-rays (0, 2, 4, 6 and 8 Gy), and colony formation assays were performed to evaluate the survival fraction. The clonogenic assay results confirmed that cells that overexpressed miR-160b were more resistant to IR than their counterparts, while miR-106b knockdown could enhance cell radiosensitivity (*p<0.05; Fig. 1b).

Ionizing radiation (IR) could trigger pro-apoptotic signals in cells with DNA damage, and the phosphorylation of histone H2AX (γ-H2AX) is an indicator of the cellular response to DNA damage [19, 20]. Immunofluorescence staining suggested that the overexpression or down-regulation of miR-106b could decrease or increase the γ-H2AX foci numbers, respectively, compared to the control groups (*p<0.05; Fig. 1c). The expression of γ-H2AX was further verified with a western blot (Fig. 1d). We also showed a similar result in the HT29 cells (Additional file 5: Figure S1C); however, the up-regulation of miR-106b did not produce significant changes in LOVO cells (data not shown). Overall, our findings documented that cells that expressed more miR-106b were more likely to be radioresistant and repair DNA damage.

In addition, we used western blots to analyse the expression of apoptosis-related genes in cells in response to irradiation. We observed that miR-106b treatment alone can decrease the expression of caspase-3, and this effect was much stronger when combined with radiation therapy (dose of X-ray 4 Gy); reducing miR-106b expression yielded the converse result (Fig. 1d). Apoptosis was assessed with flow cytometry, which showed a similar result (**P<0.01; Additional file 5: Figure S1D). Taken together, these observations illustrated a synergistic effect between miR-106b restoration and IR.

Finally, xenograft tumours of nude mice were utilized to study the sensitivity to IR. Tumour areas (dose of X-ray 8 Gy) were irradiated when the tumour size reached approximately 200 mm^3^. The tumour size was recorded every 4 days after irradiation. The results suggested that the overexpression or knockdown of miR-106b could, respectively, enhance or reduce tumour radioresistance in vivo, as demonstrated by a lower or higher tumour inhibition ratio, respectively, compared with the control groups, while down-regulation resulted in an evidently higher ratio (**p<0.01; Fig. 1e and Additional file 5: Figure S1E). We further detected the expression of Bcl-2 and Bax in xenograft tumours by immunohistochemistry and found that miR-106b could upregulate the expression of Bcl-2, while it significantly downregulated the expression of Bax (Additional file 5: Figure S1F).

### MiR-106b enhances tumour-initiating cell capacity without or with IR

**Fig. 2:**
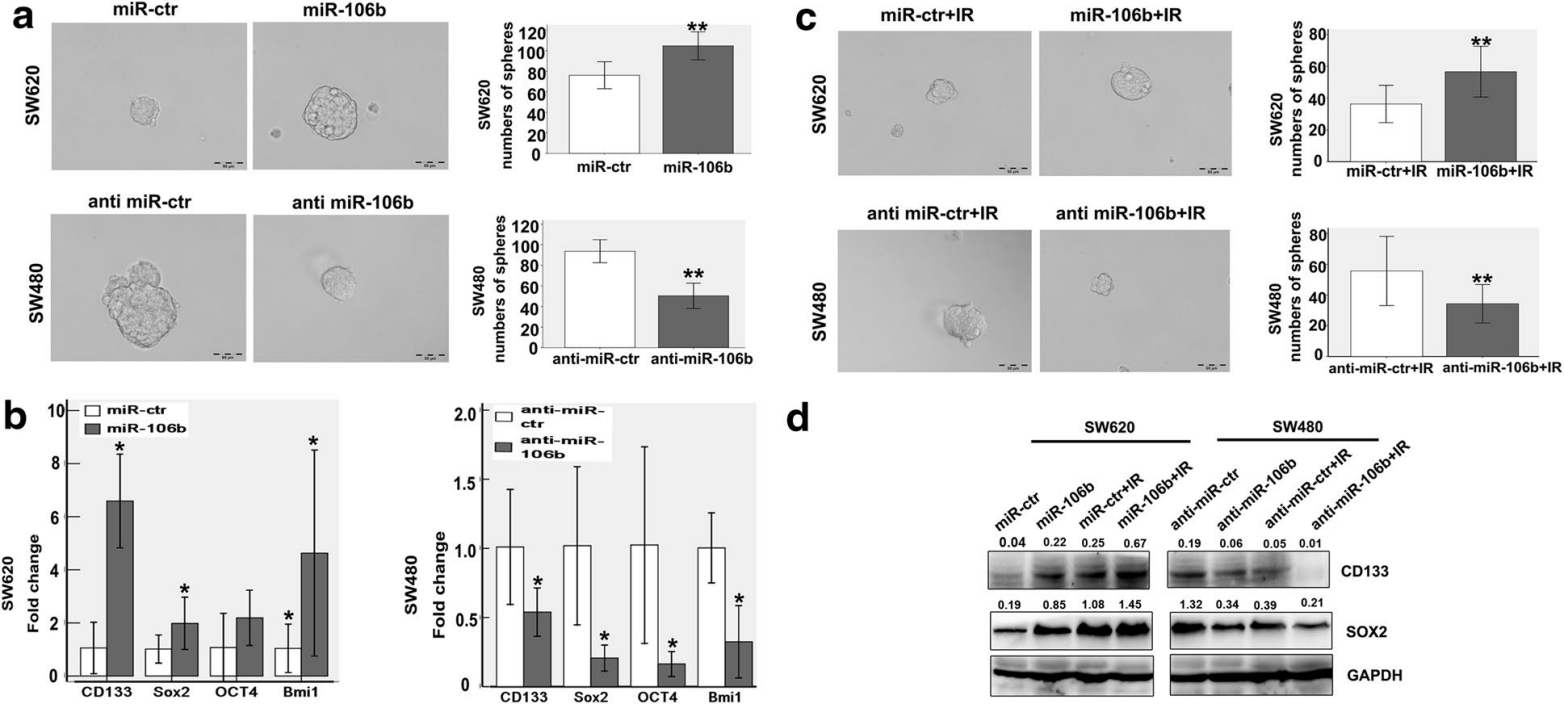
MiR-106b enhances tumour-initiating cell capacity without or with IR. **a** miR-106b induces stem cell-like self-renewal properties. The sphere sizes are shown in the *left panels*, and the numbers of spheres are shown in the *right panels*. *Scale bars* represent 50 mm. **p<0.01. **b** Genes important for stem cell maintenance, such as CD133, Sox2, oct4 and bmi1, were analysed with a qRT-PCR array. *p<0.05. **c** The ability of colorectal cancer (CRC) cell lines after exposure to radiation to form colon spheres was analysed. Sphere sizes are shown in the *left panels*, and the numbers of spheres are shown in the *right panels*. *Scale bars* represent 50 mm. **p<0.01. **d** Genes important for stem cell maintenance, i.e., CD133 and Sox2, were analysed by western blot after irradiation (4 Gy).

A colony sphere formation assay was performed to explore the effect of miR-106b on the tumour-initiating cell capacity. The data indicated that the ability of cells to form colony spheres was dramatically enhanced, as indicated by more numerous and larger spheres in cells that expressed increased levels of miR-106b. Conversely, decreasing the expression of miR-106b decreased the ability of cells to form colony spheres (**p<0.01; Fig. 2a). In addition, the expression levels stemness-related genes and proteins were detected by qRT-PCR and western blot. The levels of CD133, Sox2, Oct4 and Bmi1 were increased at the RNA level (*p<0.05; Fig. 2b). However, only CD133 and Sox2 were up-regulated at the protein level after the overexpression of miR-106b in SW620 cells (Additional file 6: Figure S2). The results were further confirmed by miR-106b knockdown in SW480 cells.

The acquisition of tumour-initiating cell capacity has been reported to be associated with tumour radioresistance. Therefore, we investigated the potential relationship between the tumour-initiating cell capacity and CRC radioresistance. The data indicated that SW620 cells that overexpressed miR-106b more readily formed colony spheres, which was accompanied by increased CD133 and Sox2 protein levels, while the inhibition of miR-106b in SW480 cells yielded the opposite effect (**p<0.01; Fig. 2c, d). However, the expression of Oct4 and Bmi1 did not show significantly alter the protein levels (data not shown).

In conclusion, cells that express high levels of miR-106b more strongly initiated tumours under both normal and IR conditions. This finding may explain why cells that express higher high levels of endogenous miR-106b exhibit greater proliferation potential and resistance to IR.

### MiR-106b targets PTEN and p21 for repression

**Fig. 3:**
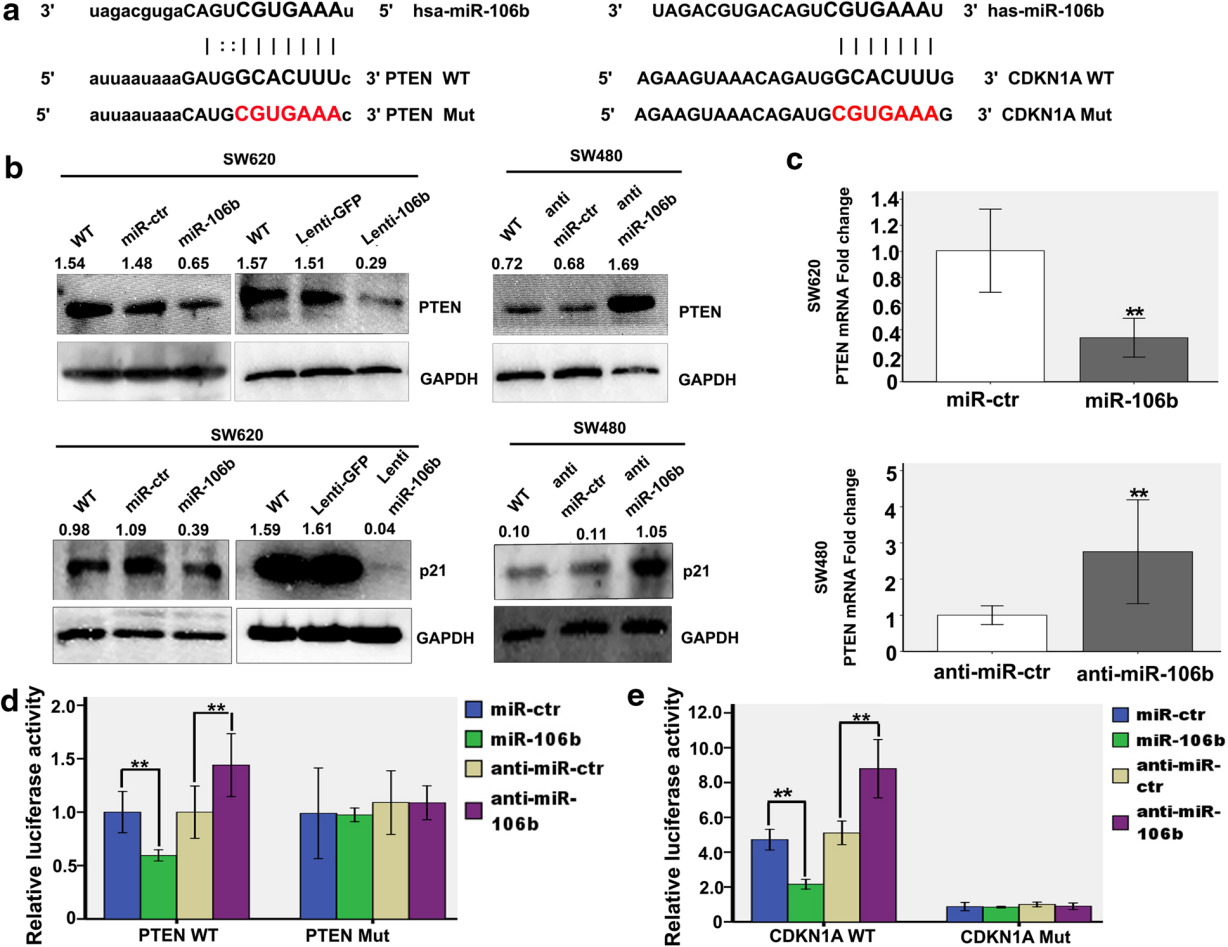
PTEN and p21 are targets of miR-106b. **a** PTEN and p21 3′UTRs contain predicted miR-106b binding sites. In the figure the alignment of the seed regions of miR-106b with PTEN and p21 3′UTRs is shown. **b** The expression levels of PTEN and p21 after the inhibition of miR-106b via lentiviral transduction in SW480 cells or the overexpression of the same miRNA by oligonucleotide transfection or lentiviral transduction in SW620 cells were detected using western blot. **c** The mRNA expression levels of PTEN after the inhibition of miR-106b in SW480 cells or the overexpression of the same miRNA in SW620 cells was detected using qRT-PCR. **p<0.01. **d** PTEN 3′UTRs are targets of miR-106b. pluc3-PTEN that contained a wild-type or mutated PTEN 3′UTRs (indicated as WT or mut on the X-axis) was transfected into SW620 or SW480 cells. The relative repression of firefly luciferase was standardized to a transfection control. The reporter assays were performed three times with essentially identical results. **p<0.01. **e** p21 3′UTRs are targets of miR-106b. pluc3-p21 that contained a wild-type p21 3′UTRs was transfected into SW620 or SW480 cells. The relative repression of firefly luciferase was standardized to a transfection control. The reporter assays were performed three times with essentially identical results.**p<0.01.

We focused on the targets of miR-106b and found via a bioinformatics search in Targetscan (http://www.targetscan.org) that the 3′-UTRs of human PTEN and p21 contained regions that matched the seed sequences of miR-106b (Fig. 3a). PTEN is an important negative regulator of PI3K-AKT signalling that is involved in the complex response to IR via the induction of cell cycle arrest in the G_2_/M phase and apoptosis [21, 22]. CDKN1A (p21), a key inhibitor of the cell cycle, is also frequently dysfunctional in human cancer [23]. Increasing the endogenous miR-106b levels by either oligonucleotide transfection (*p<0.05; Additional file 7: Figure S3A) or lentiviral transduction could significantly decrease PTEN expression both at the RNA and protein levels, but the expression of P21 was only decreased at the protein level. The inhibition of miR-106b yielded the same effect (Fig. 3b, c).

To verify whether PTEN is a direct target of miR-106b, PTEN 3′-UTR, the sequence that contains the miR-106b binding sites, was cloned into the downstream luciferase open reading frame. The co-transfection of miR-106b mimics and the PTEN-3′-UTR-wild vector into SW620 cells (pLuc-PTEN-3′-UTR) significantly decreased the luciferase activity compared with miR-NC mimics. In contrast, the transfection of miR-106b inhibitors into SW480 increased the luciferase activity. However, the transfection of mimics or inhibitors of miR-106b with the mutant 3′-UTR vector (pLuc-PTEN-mut 3′-UTR) did not affect the luciferase activity (Fig. 3d).

We also successfully constructed a reporter vector that includes the p21 3′UTR-wild vector (pLuc- p21- 3′UTR). The co-transfection of miR-106b mimics and the p21-3′UTR -wild vector into SW620 cells (pLuc-p21-3′UTR) significantly decreased the luciferase activity compared with miR-NC mimics. In contrast, the transfection of miR-106b inhibitors into SW480 significantly increased the luciferase activity (**p<0.01; Fig. 3e). The results further confirmed that PTEN and p21 are direct targets of miR-106b.

### MiR-106b mediated-radioresistance can be reversed by activating PTEN/PI3K/AKT and p21 pathway

**Fig. 4:**
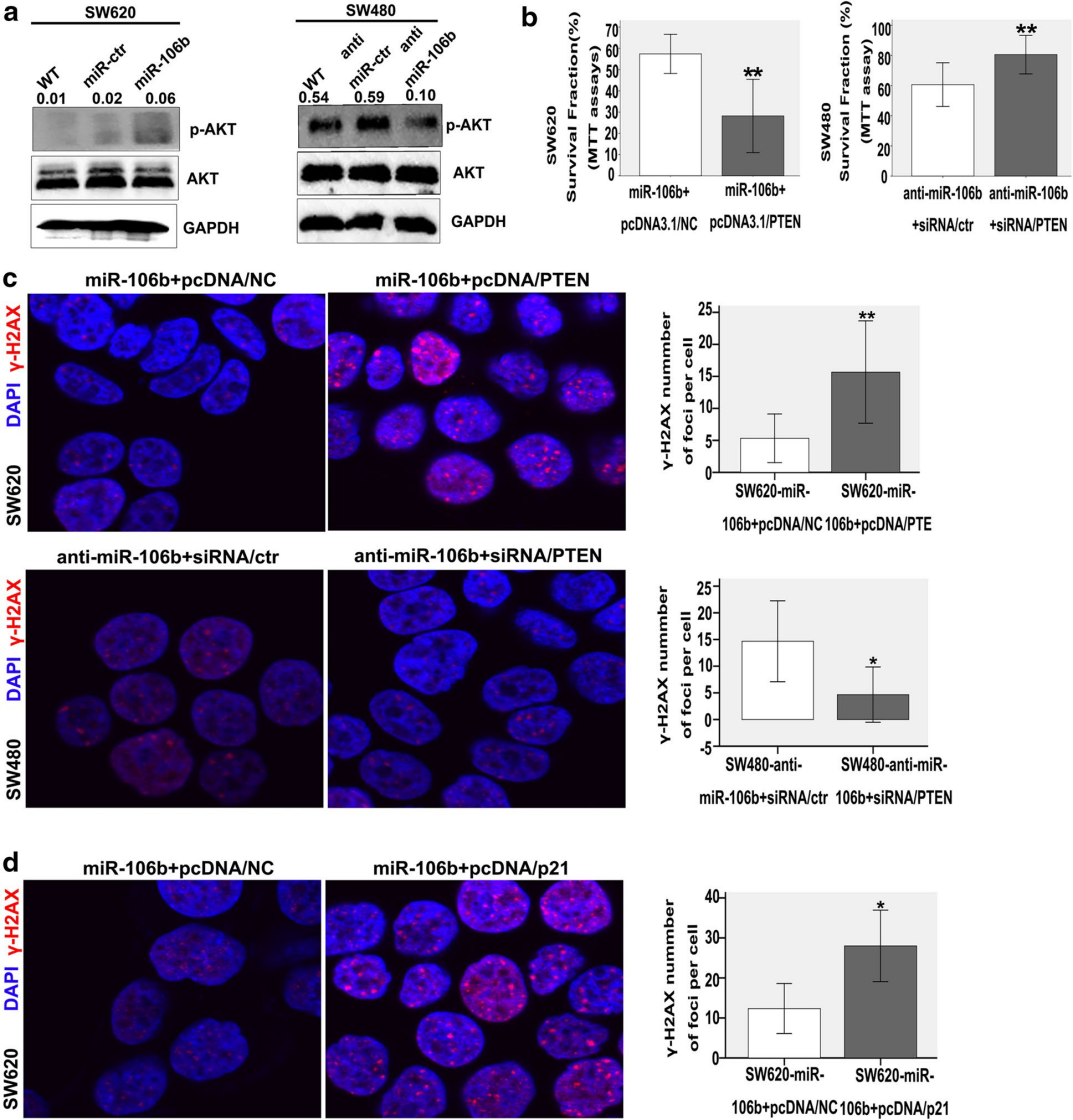
MiR-106b mediating-radioresistance can be rescued by PTEN/PI3K-AKT and p21 pathway. **a** p-AKT1/2 levels were examined by western blot after miR-106b overexpression or downregulation. **b** The detection of cell sensitivity to radiation (4 Gy) by MTT 4 days after co-transfection with mir-106b and pcDNA3.1/PTEN or anti-miR-106b and siRNA/PTEN. **p<0.01. **c** γ-H2AX was examined by immunofluorescence in the SW620 cells co-transfected with miR-106b and pcDNA3.1/PTEN or SW480 co-transfected with anti-miR-106b and siRNA/PTEN when exposed to radiation (4 Gy, 6 h). The γ-H2AX staining is shown in the *left panels*, and the numbers of γ-H2AX foci are shown in the *right panels*. **p<0.01, *p<0.05. **d** γ-H2AX was examined by immunofluorescence after co-transfection with mir-106b and pcDNA3.1/p21 in SW620 cells when exposed to radiation (4 Gy, 6 h). The γ-H2AX staining is shown in the left panels and the numbers of γ-H2AX foci are shown in the right panels. *p<0.05.

PTEN is an important negative regulator of PI3K/AKT signalling [24]. Our studies found that the overexpression of miR-106b improves p-AKT1/2 expression, and miR-106b knockdown could inhibit the expression of p-AKT1/2 (Fig. 4a).

We sought to determine whether the restoration of PTEN in miR-106b-overexpressing SW620 cells could reduce the cell resistance to IR. A pcDNA3.1/PTEN vector that expressed the PTEN gene was constructed and successfully transfected into miR-106b-overexpressing SW620 cells, and the success of this transfection was verified with a western blot (Additional file 7: Figure 3B). MTT assays showed that restoring the PTEN expression by transfecting the pcDNA3.1/PTEN vector enhanced the cell sensitivity to IR (**p<0.01; Fig. 4b). The immunofluorescence results also confirmed that the restoration of PTEN could significantly increase the number of γ-H2AX foci (**p<0.01, *p<0.05; Fig. 4c).

Additionally, the transfection of PTEN siRNA into miR-106b-knockdown SW40 cells (Additional file 7: Figure S3C) could induce cell resistance to IR, which was confirmed by MTT assays and immunofluorescence staining (*p<0.05, **p<0.01; Fig. 4b, c).

p21 is recognized to play a role in the protection of cancer cells from stress and DNA damage [25]. Our findings indicated that p21 was also a direct target of miR-106b. Thus, we sought to identify the role of p21 in cell radioresistance in the presence of miR-106b.

First, p21 was knocked down by transfecting siRNA into SW620 (Additional file 7: Figure S3D the left), and the cells were then exposed to irradiation (4 Gy). The result suggested that p21 knockdown could increase the number of γ-H2AX foci (**p<0.01; Additional file 7: Figure S3E) and the expression of γ-H2AX (Additional file 7: Figure S3F). Overall, we showed that p21 functioned as an irradiation resistance factor, which was consistent with previous studies. We then transfected the pcDNA3.1/p21 vector into miR-106b-overexpressing SW620 cells (Additional file 7: Figure S3D the right). We found that the number of γ-H2AX foci (*p<0.05; Fig. 4d) and γ-H2AX protein level were higher than those of the control group (Additional file 7: Figure S3G).

Next, we transfected siRNA/p21 into SW480-anti-miR-106b cells in order to determine whether reducing the expression of p21 in SW480-anti-miR-106b cells could restore the cell radioresistance potential. The γ-H2AX foci numbers did not significantly change compared with the control, which indicated that p21 knockdown did not affect the radioresistance under PTEN/PI3K/AKT pathway activation conditions (data not shown).

Finally, we treated miR-106b-overexpressing SW620 cells with LY294002, which is a highly selective inhibitor of Akt, and the cells were then irradiated (4 Gy). The data showed that blocking the PTEN/PI3K/AKT pathway could enhance the cell sensitivity to IR (*p<0.05; Additional file 7: Figure S3H).

Based on these data, we concluded that the PTEN/PI3K/AKT pathway plays a dominant role in miR-106b mediated-radioresistance in CRC.

### MiR-106b is inversely correlated with PTEN expression in colorectal cancer

**Fig. 5:**
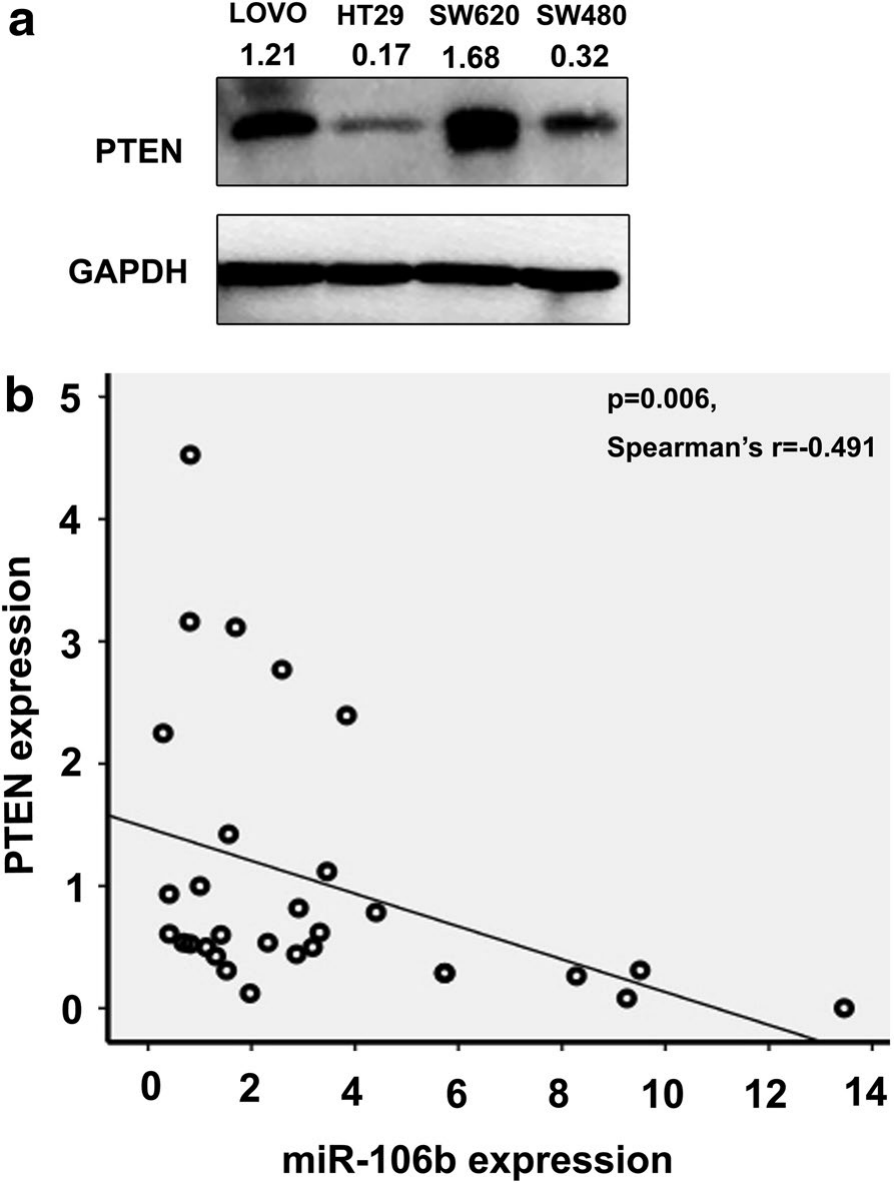
PTEN expression is inversely related to that of miR-106b in colorectal cancer. **a** A western blot for the PTEN protein was performed in colorectal cancer cell lines. **b** qRT-PCR on 15 colorectal cancer patients. The association between miR-106b and PTEN mRNA for the 15 subjects in the tumour class and for the paired 15 subjects in the normal class was statistically calculated using the Spearman correlation coefficient (p = 0.006, Spearman’s r = −0.491). The Spearman correlation indicated an inverse relation between miR-106b and PTEN mRNA in the normal and tumour samples.

We found an inverse correlation between miR-106b RNA expression and PTEN protein expression in colorectal cancer cell lines of different differentiation degrees (Fig. 5a). PTEN is one of the most frequent tumour suppressors in human cancers [26, 27], and miR-106b is upregulated in colorectal cancer tissues. Thus, we evaluated the endogenous levels of miR-106b and PTEN using real-time quantitative RT-PCR (qRT-PCR) in primary colorectal cancer tissues (Additional file 8: Figure S4). We found that the expression levels of miR-106b and PTEN significantly correlated in colorectal tissues (p = 0.006, Spearman’s r = −0.491) (Fig. 5b). The results showed that the expression of miR-106b inversely correlated with PTEN in colorectal cancer and normal colonic tissues, which further supported the finding that PTEN is a direct target of miR-106b in vivo.

## Discussion

Wang et al. previously demonstrated with a microarray that miR-106b was upregulated in colon cancer cases with lymph node metastasis [28]. Although miR-106b is clearly dysregulated in many human cancers, including CRC [29, 30], few studies are available on its roles on cancer radiosensitivity, especially in CRC.

Highly differentiated adenocarcinoma is commonly more resistant to irradiation than poorly differentiated adenocarcinoma. HT-29 and SW480 are known as highly differentiated cell lines with high levels of endogenous miR-106b. The expression of this miR-106b is low in the SW620 cell line, which is poorly differentiated. Therefore, we selected the SW620 and SW480 cell lines for the subsequent gain and loss of function studies, respectively. The overexpression of miR-106b could inhibit cell apoptosis, promote proliferation and induce radioresistance in vitro and in vivo when cells were irradiated, which revealed that miR-106b may be an anti-apoptotic and DNA damage repair factor in the presence of radiation.

p21 is a well-known tumour suppressor that functions by mediating G1 growth arrest and cellular senescence [31], and previous studies also revealed that p21 knockdown was closely related to metastasis and poor survival in CRC [32, 33]. We have verified that p21 is a direct target of miR-106b, as confirmed by a bioinformatics search and a luciferase assay. We also found that miR-106b could induce G1 to S transition (Additional file 9: Figure S5), which is a radioresistant cell cycle phase [34]. In summary, p21 may play an important role in the miR-106b-mediated G1 to S transition and radioresistance, which may also explain why cells that express high levels of endogenous miR-106b are highly resistant to IR.

Previous studies of different tumours confirmed that p21 is closely linked to a favourable prognosis [31, 33]. However, p21 protects cells from death following anticancer treatments, including exposure to radiation, which has been confirmed over the past 30 years [22, 35, 36]. Based on these previous studies, the function of p21 appears to be pleiotropic. Our results determined that at a “relatively normal level” of miR-106b cells (such as that in wild type cells, like SW620), p21 knockdown could enhance cell sensitivity to IR, which indicated that p21 functioned as a DNA damage repair factor. This result was consistent with those of previous studies. However, we also found that p21 functioned as a DNA damage factor, not a repair factor, when the expression of miR-106b was up- or downregulated. The overexpression of p21 in miR-106b-overexpressing SW620 cells could reverse the miR-106b-mediated radioresistance, which suggested that p21 functioned as a DNA damage repair inhibitor. We verified that p21 had little function in DNA damage repair at high levels of miR-106b and activated PI3K-AKT conditions; inversely, it induced growth arrest and senescence and functioned as a DNA damage repair inhibitor.

Thus, we inferred that p21 played dual roles in the DNA damage repair process, as a promoter or inhibitor, and this role depended on its cellular context. Moreover, miR-106b may be one of the important factors that affect this role. Priority should be given to block p21 in order to promote DNA damage repair. This approach allows us to more effectively utilize p21 as a therapeutic target that preferentially preserves its tumour suppressive functions, such as growth arrest.

Our research elucidated that miR-106b could enhance the cell self-renew capacity and upregulate the expression of stem cell markers under both normal and radiation conditions. Previous studies have shown that both normal and cancer stem cells are chemoresistant and radioresistant [37]. We provide a direct link between the self-renewal capacity and radioresistance, which may partially explain why the overexpression of miR-106b could induce cell radioresistance. p21 played dual roles in stem cells; for example, basal p21 expression levels maintain stem cells quiescence, while increasing p21 expression promotes cells differentiation and limits the self-renewal potential [34]. When miR-106b increases the capacity of cells to initiate tumours, increased p21 expression promotes cell differentiation and limits self-renewal potential, which may partially explain why p21 minimally impacts the repair of DNA damage caused by miR-106b.

By integrating a bioinformatics search (Targetscan) and luciferase assay, we identified PTEN as another functional direct target of miR-106b. Studies have shown that PTEN plays an important role in both the early and late stages of CRC and PTEN loss positively correlated with malignant progression, such as tumour size and the TNM advanced stage [38]. Recent studies showed that the restoration of PTEN could decrease the ratio of metastases in an orthotopic model of colon cancer [39].

We demonstrated that the induction of miR-106b expression could produce a radioresistant phenotype in the normally radiosensitive SW620 cells, while the restoration of PTEN could partially reverse this effect. Similarly, PTEN knockdown could restore the radioresistance of SW480, which may have been due to miR-106b downregulation. To the best of our knowledge, PTEN is a natural inhibitor of PI3K at the 3-phosphate site and negatively regulates the AKT signalling pathway [40]. We proved that miR-106b could positively regulate p-AKT expression by downregulating PTEN. Blocking the PI3K/AKT pathway by using LY294002 could reverse the miR-106b-mediated radioresistance. Based on the above results, we concluded that miR-106b enhanced the cell resistance to IR via PTEN/PI3K/AKT pathway.

Dubrovska et al. found that the PTEN/PI3K/Akt pathways are critical for prostate cancer stem-like cell maintenance and that targeting PI3K signalling may be beneficial in prostate cancer treatment by eliminating prostate cancer stem-like cells [41]. However, we found that p21 and PTEN indirectly interact with CD133 or sox2. Thus, we hypothesise that p21 and PTEN interact with CD133 or sox2 via some other genes or signalling pathways.

The PI3K-AKT pathway can undoubtedly be activated by miR-106b via the direct targeting of PTEN. The restoration of p21 and PTEN expression or treatment with a PI3K-AKT pathway inhibitor can reverse miR-106b-induced radioresistance. However, p21 knockdown in SW480- anti-miR-106b cells did not induce significant radiosensitivity changes, which indicated that p21 downregulation insufficiently reversed the radiosensitivity and did not ameliorate the DNA damage status, unless the PTEN/PI3K-AKT pathway was activated by miR-106b knockdown. We speculated that p21 is a point of internal contact and interaction between two genes and that the PTEN/PI3K-AKT pathway plays a dominant role during p21 function transformation and miR-106b-mediated cell radioresistance in CRC.

To further assess the relationship between PTEN and miR-106b, we screened the PTEN expression in CRC cell lines as well as clinical specimens. Our data indicated that HT29 and SW480 cells, which express higher levels of endogenous miR-106b and have been verified as radioresistant [42], expressed less PTEN than the more radiosensitive cell lines SW620 and LOVO cells. The clinical specimens also revealed that elevated levels of miR-106b correlated with low PTEN expression, indicating miR-106b may be a causal factor for PTEN loss in CRC.

## Conclusion

**Fig. 6:**
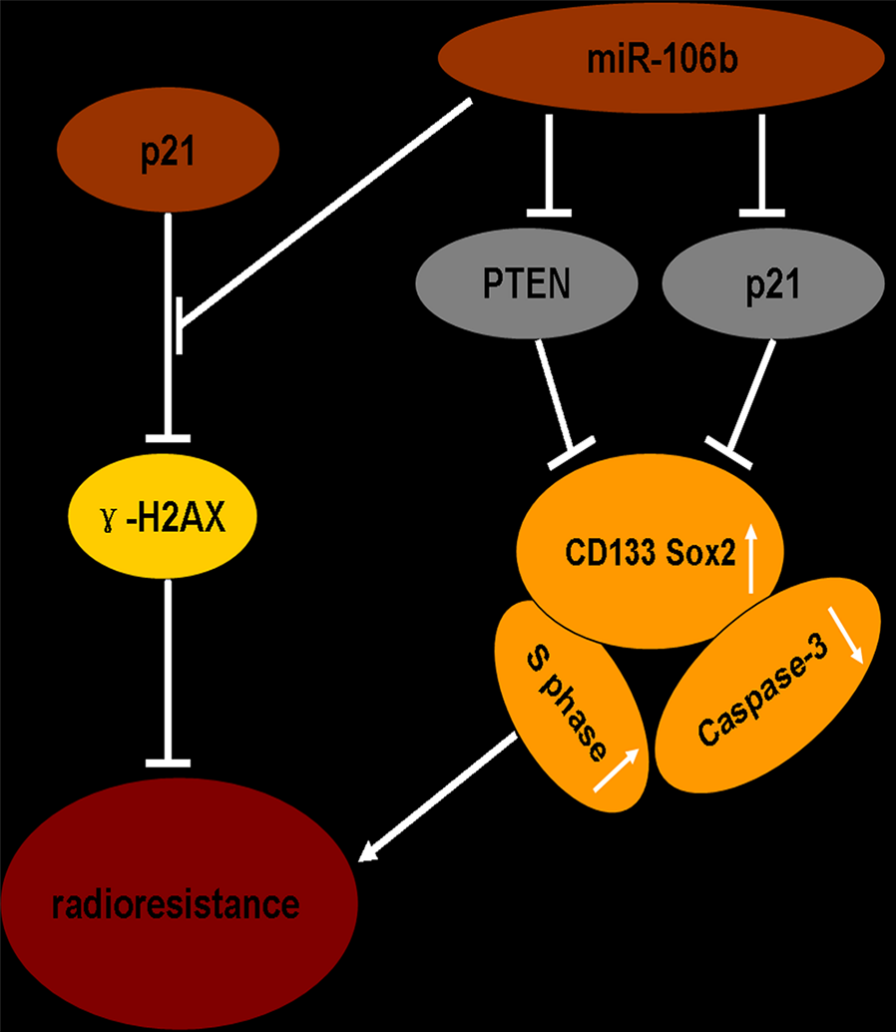
MiR-106b enhances radioresistance by targeting PTEN and p21. miR-106b upregulation downregulates PTEN and p21 and subsequently enhances radioresistance. p21 functioned as a DNA damage repair promoter at “a relatively normal level” of miR-106b and PI3K/AKT, while it acted as a DNA damage repair inhibitor when altering miR-106b expression.

In this study, we identified miR-106b could induce cell radioresistance by targeting p21, which induces G_1_/S transition and restrains apoptosis and activating the PTEN/PI3K-AKT pathway. Moreover, this process was accompanied by an enhancement of the tumour-initiating cell capacity, suggesting that miR-106b may account for the resistance to radiotherapy and may be a potential clinical therapeutic target for patients who rarely benefit from radiation therapy in CRC (Fig. 6).

## Additional files

Additional file 1:
**Table S1.** Primers for quantification. Additional file 2: Construction of PTEN and p21 vectors and transfection. Additional file 3: miR-106b overexpression  or knockdown amounts of miR-106b were established via lentiviral transduction. Additional file 4: Construction of luciferase reporter vectors. Additional file 5: MiR-106b induces CRC cell resistance to irradiation. **Figure S1.**  (A) The expression pattern of miR-106b in CRC cell lines with different degrees of differentiation. The relative expression of miR-106b was normalized to the endogenous control U6. (B) The up-regulation or inhibition of miR-106b in SW620 via lentiviral transduction in SW480 cells was detected using qRT-PCR. *p<0.05. (C) γ-H2AX was examined by immunofluorescence in the HT-29 cells transfected with miR-106b when exposed to radiation (4 Gy, 6 h). The γ-H2AX staining is shown in the left panels, and the numbers ofγ-H2AX foci are shown in the right panels. *p<0.05. (D) Apoptosis as evaluated by flow cytometry when exposed to radiation (4 Gy). **P<0.005, **P<0.01. (E) Photographs of tumours from mice injected with cells. (F) The expression of Bcl-2 and Bax in xenograft tumours exposed to radiation was detected by immunohistochemistry. Additional file 6: Detection of stemness-related markers. **Figure S2.** Genes important for stem cell maintenance, i.e., CD133 and Sox2, were examined by western blot after miR-106b overexpression or downregulation. Additional file 7: MiR-106b mediated-radioresistance can be reversed by activating PTEN/PI3K/AKT and p21 pathway. **Figure S3.** (A) The mRNA expression levels of miR-106b in SW620 cells after the forced expression of miR-106b by oligonucleotide transfection was detected using qRT-PCR *p<0.05. (B) and (C) PTEN protein expression in SW480 cells co-transfected with miR-106b and pcDNA3.1/PTEN in SW620 or co-transfected with anti-miR-106b and siRNA/PTEN. (D) The p21 protein expression was examined by western blot in SW620 cells transfected with siRNA/p21 or co-transfected with miR-106b and pcDNA3.1/p21. (E) γ-H2AX was examined by immunofluorescence after transfection with siRNA/p21 in SW620 cells. The γ-H2AX staining is shown in the left panels, and the numbers of γ-H2AX foci are shown in the right panels. **p<0.01. (F) The γ-H2AX protein expression was examined by western blot in SW620 cells transfected with siRNA/P21. (G) The γ-H2AX protein expression was examined by western blot in SW620 cells co-transfected with miR-106b and pcDNA3.1/p21. (H) γ-H2AX was examined by immunofluorescence in the SW620-miR-106b cells treated with LY294002. The γ-H2AX staining is shown in the left panels, and the numbers of γ-H2AX foci are shown in the right panels. *p<0.05. Additional file 8: qRT-PCR of samples from 15 colorectal cancer patients. **Figure S4.** The expression of miR-106b and PTEN mRNA was detected by qRT-PCR in the 15 subjects from the tumour group and the 15 paired normal controls. Additional file 9: MiR-106b promotes G1 to S transition. **Figure S5.** Comparison of G1/S fractions after miR-106b overexpression in SW620 cells or downregulation in SW480 cells by flow cytometry. The percentage of cells in the G1 and S phases and the statistic analysis are shown in the right panel. **p<0.01.

## Abbreviations

CRC: colorectal cancer
PTEN: phosphatase and tensinhomolog
3′UTR: 3-untranslated Regions
IR: ionizing radiation
γ-H2AX: phosphorylation of histone H2AX
NSPC: stem/progenitor cells
NSC: neural stem cells
