# Supplementary material for: MiR-106b induces cell radioresistance via the PTEN/PI3K/AKT pathways and p21 in colorectal cancer
Source: J Transl Med. 2015 Aug 4;13:252. doi: 10.1186/s12967-015-0592-z (PMC4522974; doi:10.1186/s12967-015-0592-z)
Supplement: Additional file 1: — Table S1. Primers for quantification. [file 12967_2015_592_MOESM1_ESM.doc]

**Table S1. Primers for quantification.**

| Name | Sequence(5’-3’) |
| --- | --- |
| Oct4-F | ACCGAGTGAGAGGCAACC |
| Oct4-R | TGAGAAAGGAGACCCAGCAG |
| Sox2-F | CGAGTGGAAACTTTTGTCGGA |
| Sox2-R | TGTGCAGCGCTCGCAG |
| Bmi1-F | AAATGCTGGAGAACTGGAAAG |
| Bmi1-R | CTGTGGATGAGGAGACTGC |
| CD133-F | TGGATGCAGAACTTGACAACGT |
| CD133-R | ATACCTGCTACGACAGTCGTGGT |
| U6-F | CGCTTCGGCAGCACATATAC |
| U6-R | CAGGGGCCATGCTAATCTT |
| GAPDH-F | GGAGCGAGATCCCTCCAAAAT |
| GAPDH-R | GGCTGTTGTCATACTTCTCATGG |
| miR-106b-RT | GTCGTATCCAGTGCAGGGTCCGAGGTATTCGCACTGGATACGACATCTG |
| miR-106b-F | TGTAAAGTGCTGACAGTGCA |
| miR-106b-R | GTGCAGGGTCCGAGGTATTC |
