# Supplementary figures and images for: MiR-106b induces cell radioresistance via the PTEN/PI3K/AKT pathways and p21 in colorectal cancer
Source: J Transl Med. 2015 Aug 4;13:252. doi: 10.1186/s12967-015-0592-z (PMC4522974; doi:10.1186/s12967-015-0592-z)

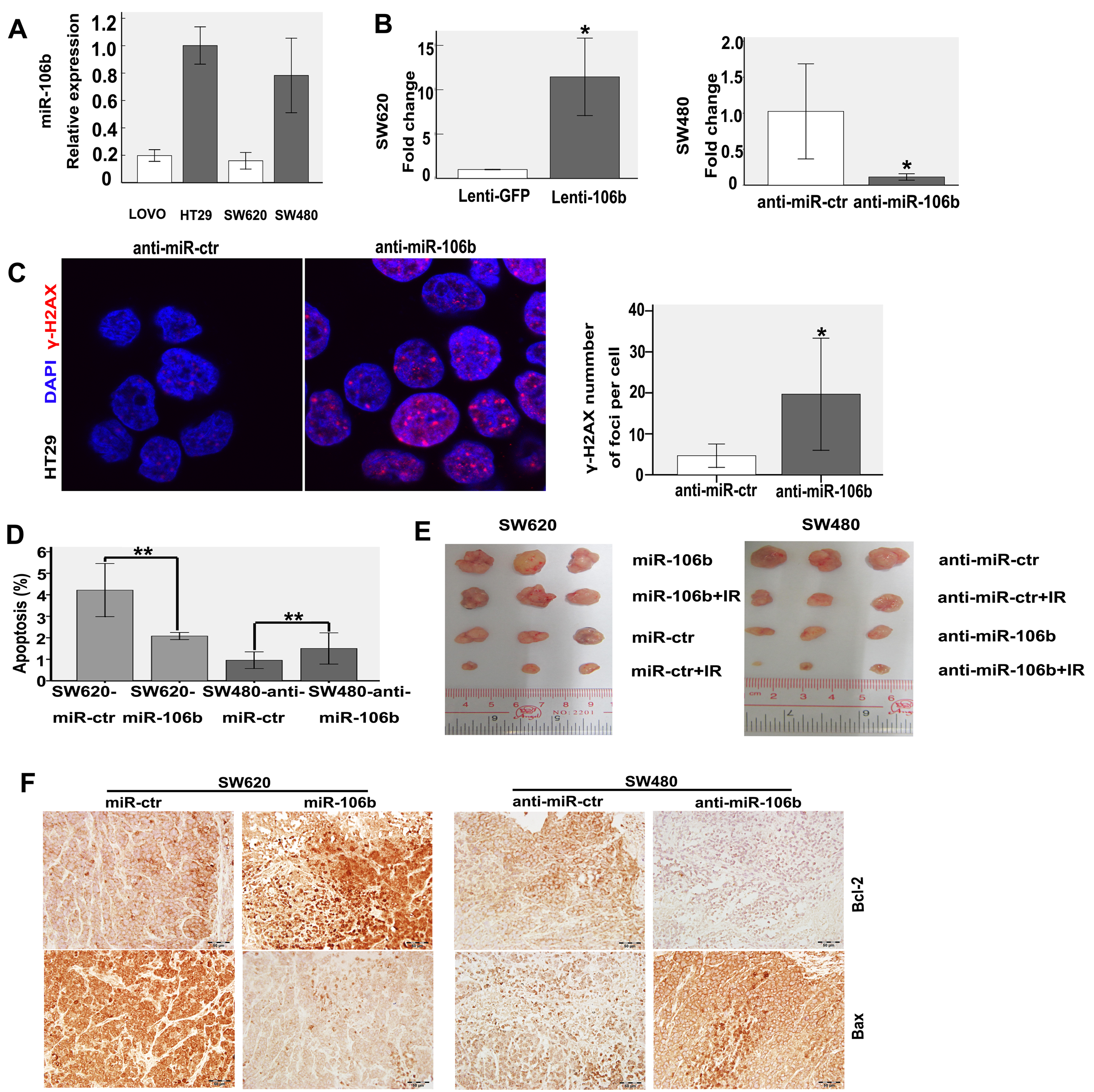

Supplement: Additional file 5: — MiR-106b induces CRC cell resistance to irradiation. Figure S1. (A) The expression pattern of miR-106b in CRC cell lines with different degrees of differentiation. The relative expression of miR-106b was normalized to the endogenous control U6. (B) The up-regulation or inhibition of miR-106b in SW620 via lentiviral transduction in SW480 cells was detected using qRT-PCR. *p<0.05. (C) γ-H2AX was examined by immunofluorescence in the HT-29 cells transfected with miR-106b when exposed to radiation (4 Gy, 6 h). The γ-H2AX staining is shown in the left panels, and the numbers ofγ-H2AX foci are shown in the right panels. *p<0.05. (D) Apoptosis as evaluated by flow cytometry when exposed to radiation (4 Gy). **P<0.005, **P<0.01. (E) Photographs of tumours from mice injected with cells. (F) The expression of Bcl-2 and Bax in xenograft tumours exposed to radiation was detected by immunohistochemistry. [file 12967_2015_592_MOESM5_ESM.tiff]

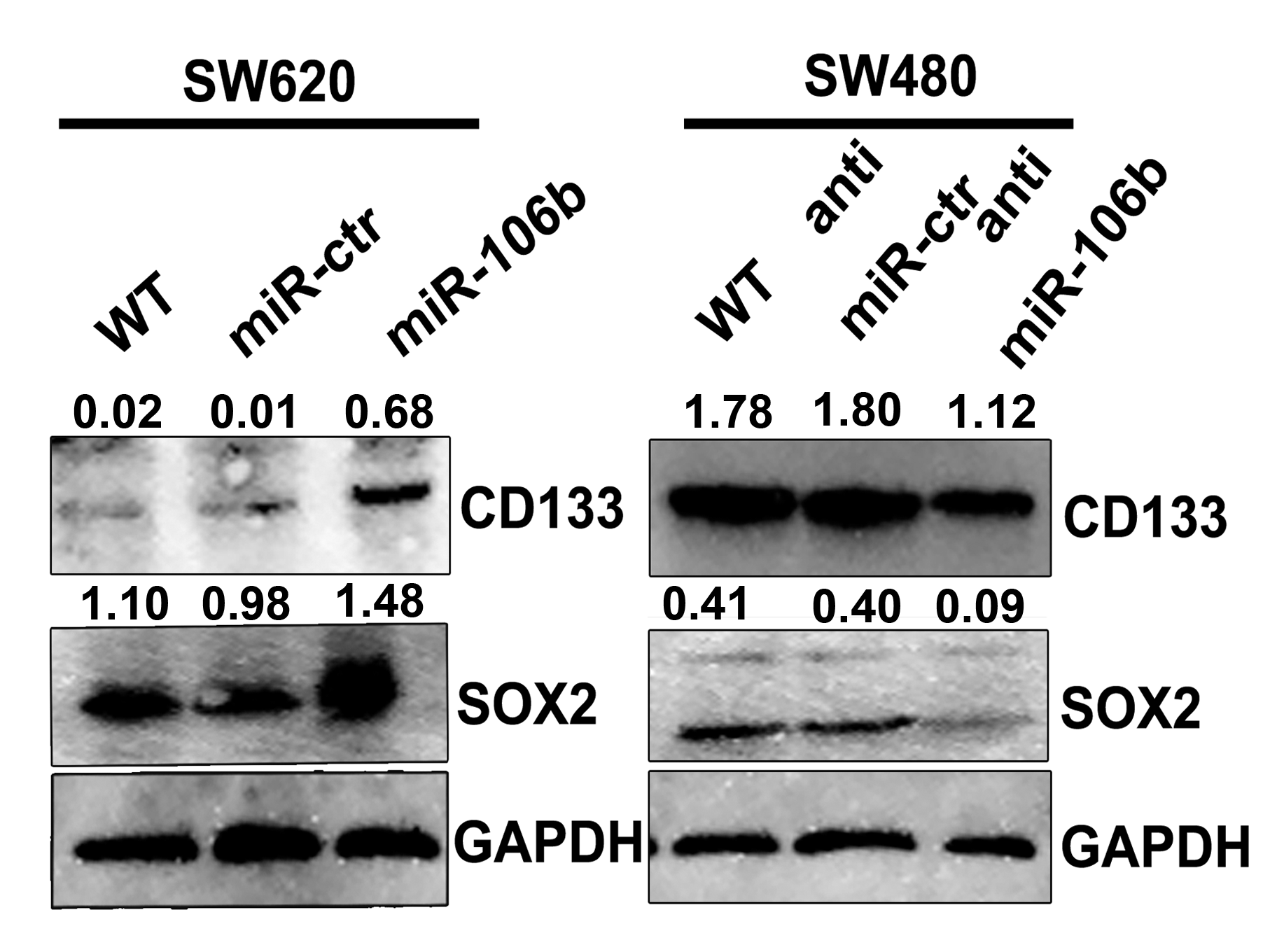

Supplement: Additional file 6: — Detection of stemness-related markers. Figure S2. Genes important for stem cell maintenance, i.e., CD133 and Sox2, were examined by western blot after miR-106b overexpression or downregulation. [file 12967_2015_592_MOESM6_ESM.tiff]

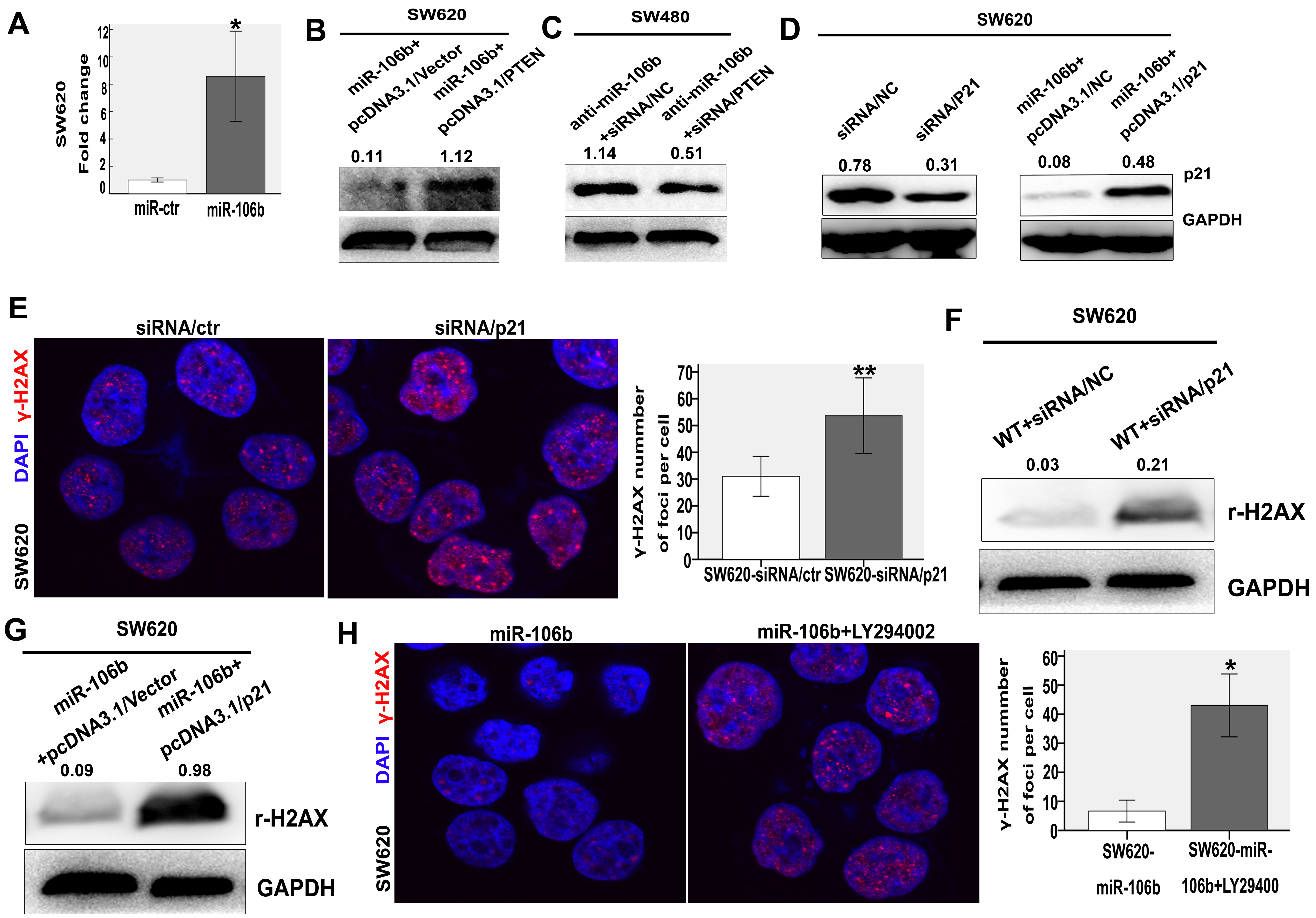

Supplement: Additional file 7: — MiR-106b mediated-radioresistance can be reversed by activating PTEN/PI3K/AKT and p21 pathway. Figure S3. (A) The mRNA expression levels of miR-106b in SW620 cells after the forced expression of miR-106b by oligonucleotide transfection was detected using qRT-PCR *p<0.05. (B) and (C) PTEN protein expression in SW480 cells co-transfected with miR-106b and pcDNA3.1/PTEN in SW620 or co-transfected with anti-miR-106b and siRNA/PTEN. (D) The p21 protein expression was examined by western blot in SW620 cells transfected with siRNA/p21 or co-transfected with miR-106b and pcDNA3.1/p21. (E) γ-H2AX was examined by immunofluorescence after transfection with siRNA/p21 in SW620 cells. The γ-H2AX staining is shown in the left panels, and the numbers of γ-H2AX foci are shown in the right panels. **p<0.01. (F) The γ-H2AX protein expression was examined by western blot in SW620 cells transfected with siRNA/P21. (G) The γ-H2AX protein expression was examined by western blot in SW620 cells co-transfected with miR-106b and pcDNA3.1/p21. (H) γ-H2AX was examined by immunofluorescence in the SW620-miR-106b cells treated with LY294002. The γ-H2AX staining is shown in the left panels, and the numbers of γ-H2AX foci are shown in the right panels. *p<0.05. [file 12967_2015_592_MOESM7_ESM.tiff]

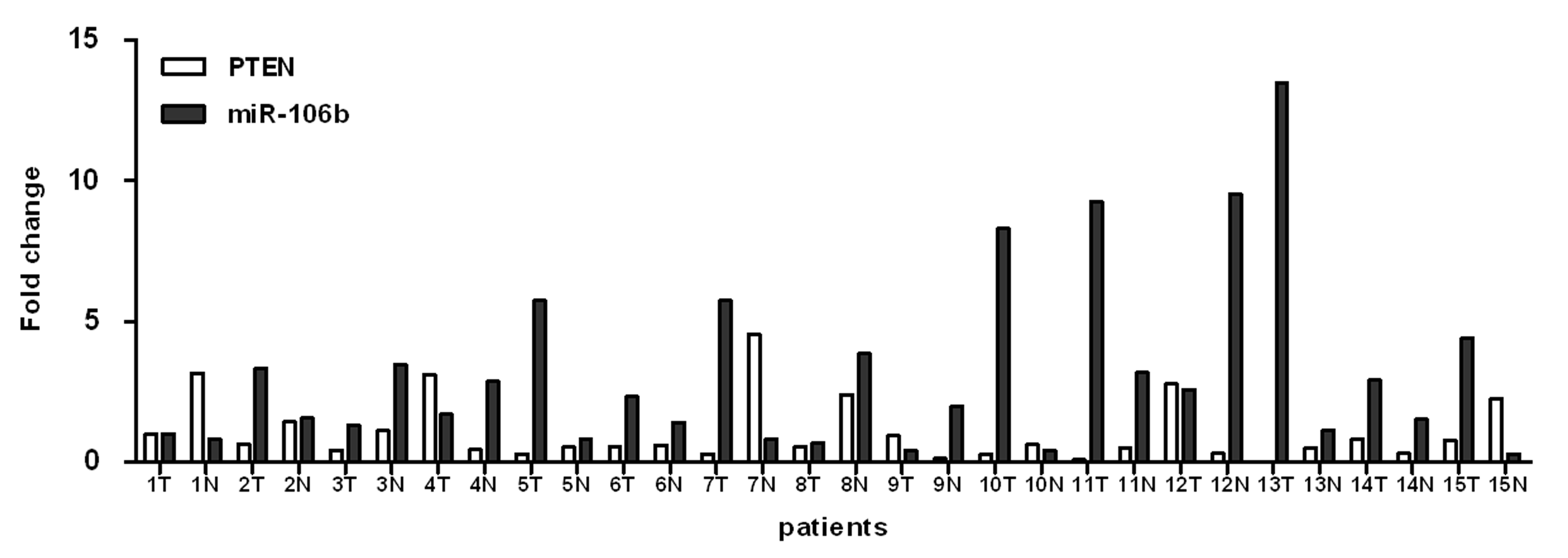

Supplement: Additional file 8: — qRT-PCR of samples from 15 colorectal cancer patients. Figure S4. The expression of miR-106b and PTEN mRNA was detected by qRT-PCR in the 15 subjects from the tumour group and the 15 paired normal controls. [file 12967_2015_592_MOESM8_ESM.tiff]

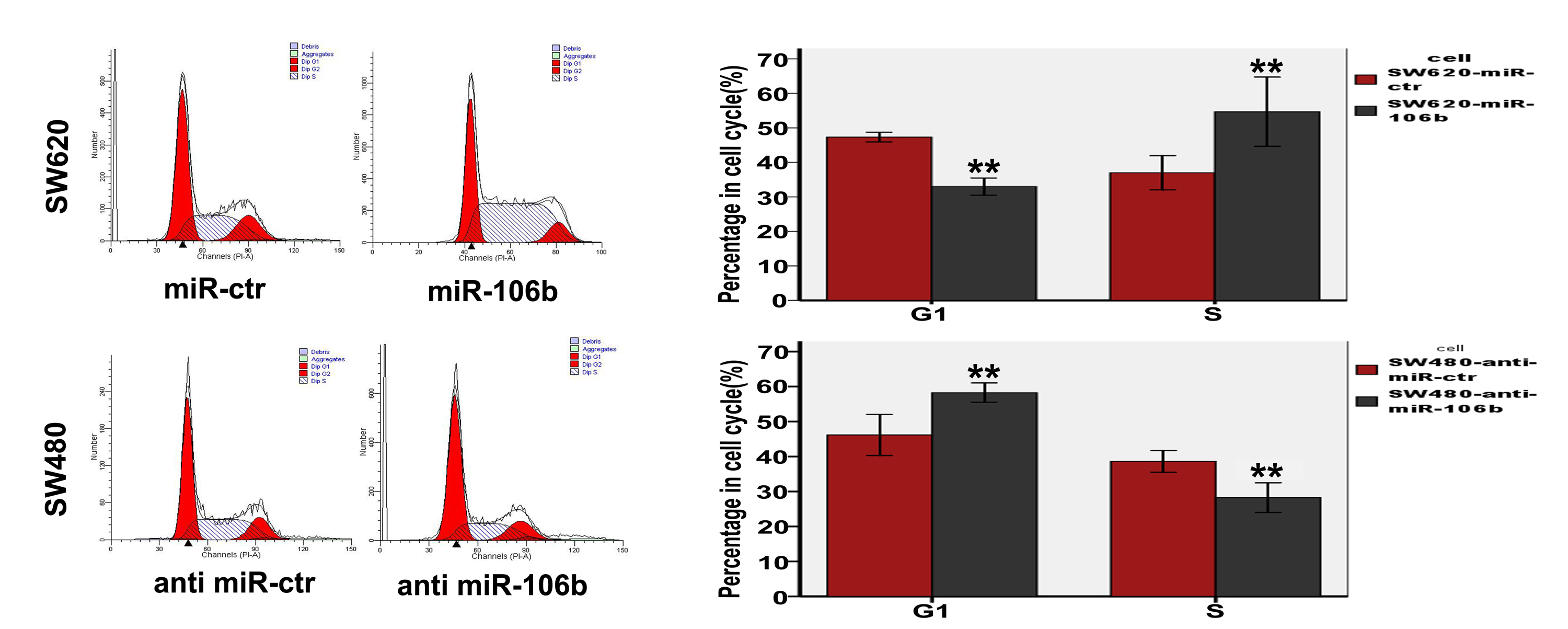

Supplement: Additional file 9: — MiR-106b promotes G1 to S transition. Figure S5. Comparison of G1/S fractions after miR-106b overexpression in SW620 cells or downregulation in SW480 cells by flow cytometry. The percentage of cells in the G1 and S phases and the statistic analysis are shown in the right panel. **p<0.01. [file 12967_2015_592_MOESM9_ESM.tiff]
